# Supplementary material for: Pushing the limits of de novo genome assembly for complex prokaryotic genomes harboring very long, near identical repeats
Source: Nucleic Acids Res. 2018 Aug 23;46(17):8953–65. doi: 10.1093/nar/gky726 (PMC6158609; doi:10.1093/nar/gky726)
Supplement: Supplementary Data [file gky726_supplemental_files.zip › Supplementary_Figures_and_Tables_revised_notmarked.pdf]

Supplementary Material

Pushing the limits of *de novo* genome assembly for complex prokaryotic genomes harboring very long, near identical repeats

Michael Schmid[1,2], Daniel Frei[1], Andrea Patrignani[3], Ralph Schlapbach[3], Jürg E. Frey[1], Mitja N.P. Remus-Emsermann[4,5], Christian H. Ahrens[1,2#]

[1]Agroscope, Research Group Molecular Diagnostics, Genomics & Bioinformatics, CH-8820 Wädenswil, Switzerland
[2]SIB Swiss Institute of Bioinformatics, CH-8820 Wädenswil, Switzerland
[3]Functional Genomics Center Zurich, CH-8057 Zurich, Switzerland
[4]School of Biological Sciences, University of Canterbury, 8140 Christchurch, New Zealand
[5]Biomolecular Interaction Centre, University of Canterbury, 8140 Christchurch, New Zealand

Table of Contents

1. Supplementary Figure S1 2
2. Supplementary Figure S2 3
3. Supplementary Figure S3 4
4. Supplementary Figure S4 5
5. Supplementary Table S1 6
6. Supplementary Table S2 7
7. Supplementary Table S3 8
8. Supplementary Code Listing 9
9. Supplementary Table S4 9
10. Supplementary Table S5 10

1. Supplementary Figure S1

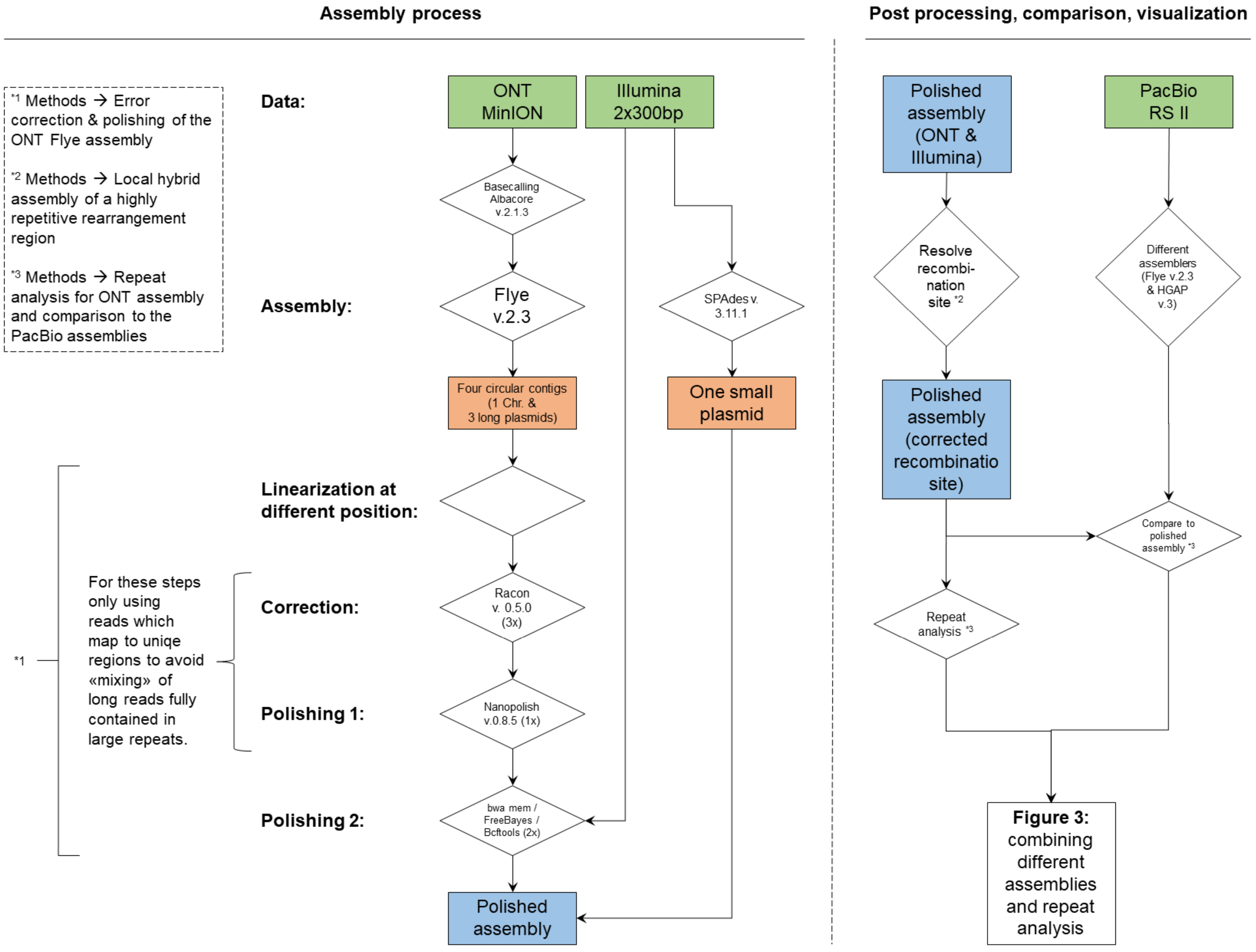

Supplementary Figure S1. Overview of workflow to create the final assembly, carry out error correction, and comparison to previous assemblies.

2. Supplementary Figure S2

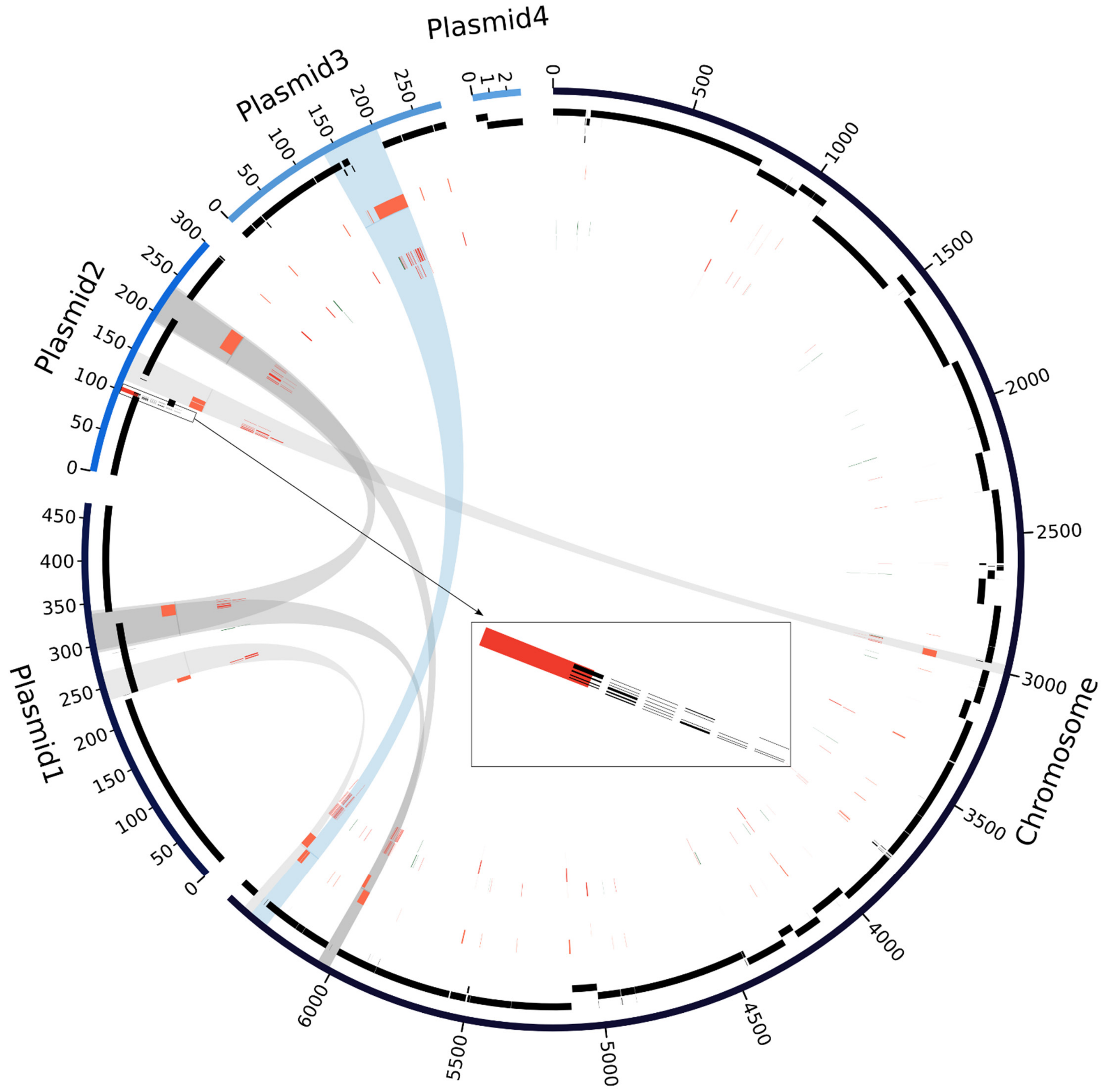

Genome assembly comparison

	Contigs	Assembly size (bp)	Missed genomic regions (bp)	# genes (compared to NCBI annotation)	# genes missed / disrupted
ONT assembly	5	7,498,194	-	7141	-
Illumina assembly	159	7,225,269	297,968*	6919 (96.9%)	222

Supplementary Figure S2. Comparison of the final high-quality *P. koreensis* P19E3 genome assembly with an assembly based only on Illumina reads. For display, the five circular elements (one chromosome, 4 plasmids) were linearized and not drawn to scale. Going from outward to inward circles: 1) ONT data assembly using Flye, 2) Illumina data assembled into 159 contigs with SPAdes, 3) Regions that are missing entirely in the Illumina assembly (orange tracks) and 4) position of genes that are either missed (orange) or disrupted (green) in the Illumina assembly. As in Figure 3, repeats above 30 kb are shown in the center (blue and gray bands, blue showing the longest repeat); they coincide with areas where the Illumina-based assembly misses parts of the genome. The genomic region harboring the shufflon (ca. 5 kb) is also shown (red mark on plasmid 2). The box shows a zoom in for the shufflon region at a high resolution to highlight how fragmented the Illumina assembly is in this specific area. A table summarizing the assembly differences is shown on the right side.

* As the Illumina assembly is partially overlapping, a total of 297,968 base pairs are missing (i.e., more than the 272,925 bp missing when comparing the total amount of Illumina bp versus the ONT size of the assembly); this corresponds to 3.97% of the ONT genome size.

3. Supplementary Figure S3

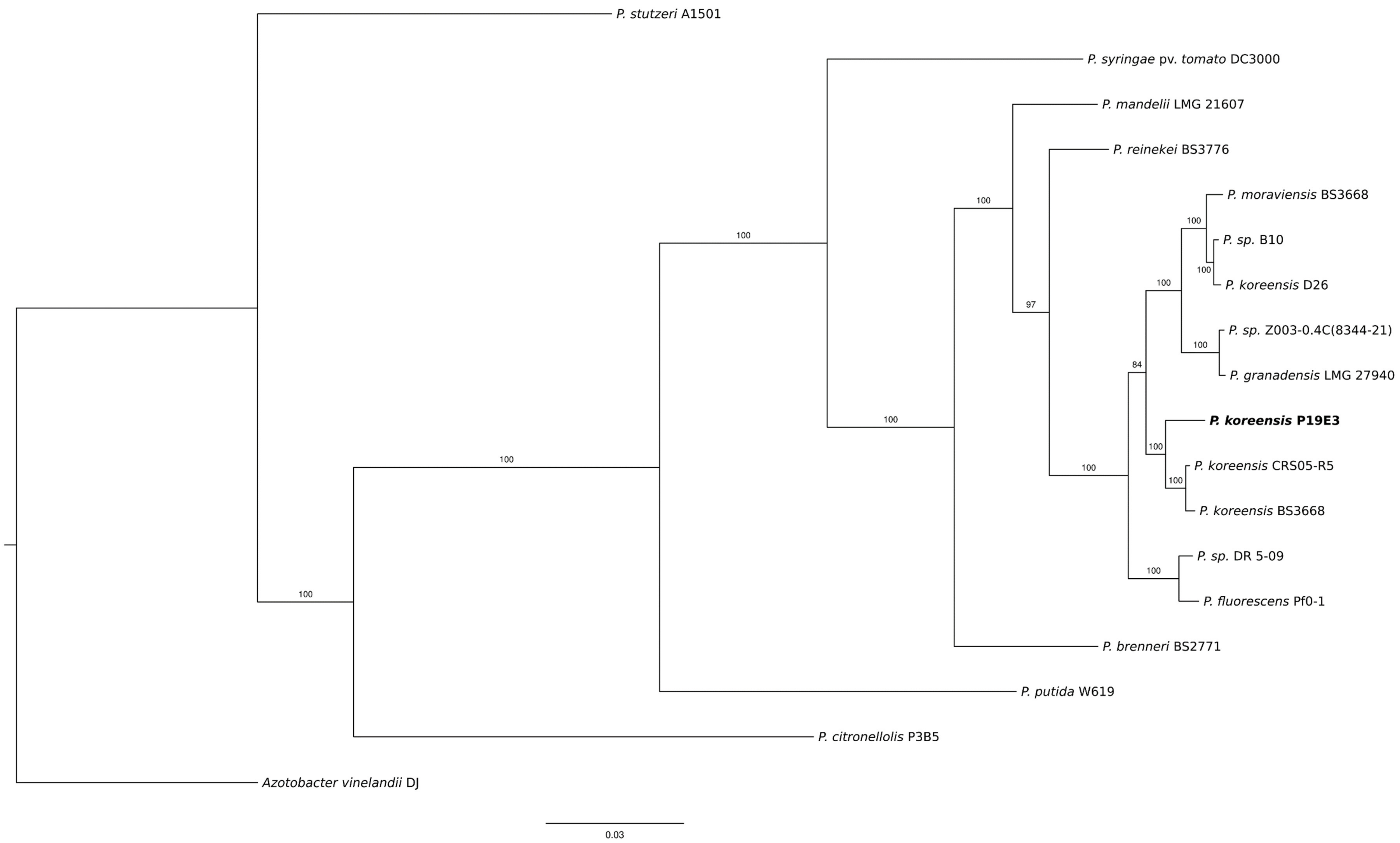

Supplementary Figure S3. Maximum likelihood phylogenetic tree placing P. koreensis P19E3 in context. P19E3 is shown in bold and it is grouped into a clade of three P. koreensis strains. The phylogenetic tree was constructed using amino acid sequence alignment of 107 housekeeping genes. Bootstrap support is shown for all nodes (100 bootstrap runs). The bar at the bottom reflects the number of amino acid changes per site. Azotobacter vinelandii DJ served as outgroup. This analysis also contained genomes with assembly level "Chromosome" since this analysis only dealt with phylogenetic aspects, not with assembly complexity. The NCBI GenBank/RefSeq accession numbers are as follows: Azotobacter vinelandii DJ: NC_012560; Pseudomonas brenneri BS2771: NZ_LT629800; Pseudomonas citronellolis P3B5: NZ_CP014158; Pseudomonas fluorescens Pf0-1: NC_007492; Pseudomonas granadensis LMG 27940: NZ_LT629778; Pseudomonas koreensis BS3658: NZ_LT629687; Pseudomonas koreensis CRS05-R5: NZ_CP015852; Pseudomonas koreensis D26: NZ_CP014947; Pseudomonas koreensis P19E3: CP027477; Pseudomonas mandelii LMG 21607: NZ_LT629796; Pseudomonas moraviensis BS3668: NZ_LT629788; Pseudomonas putida W619: NC_010501; Pseudomonas reinekei BS3776: NZ_LT629709; Pseudomonas sp. B10: NZ_LT707063; Pseudomonas sp. DR 5-09: NZ_CP011566; Pseudomonas sp. Z003-0.4C(8344-21): NZ_LT629756; Pseudomonas stutzeri A1501: NC_009434; Pseudomonas syringae pv. tomato DC3000: NC_004578.

4. Supplementary Figure S4

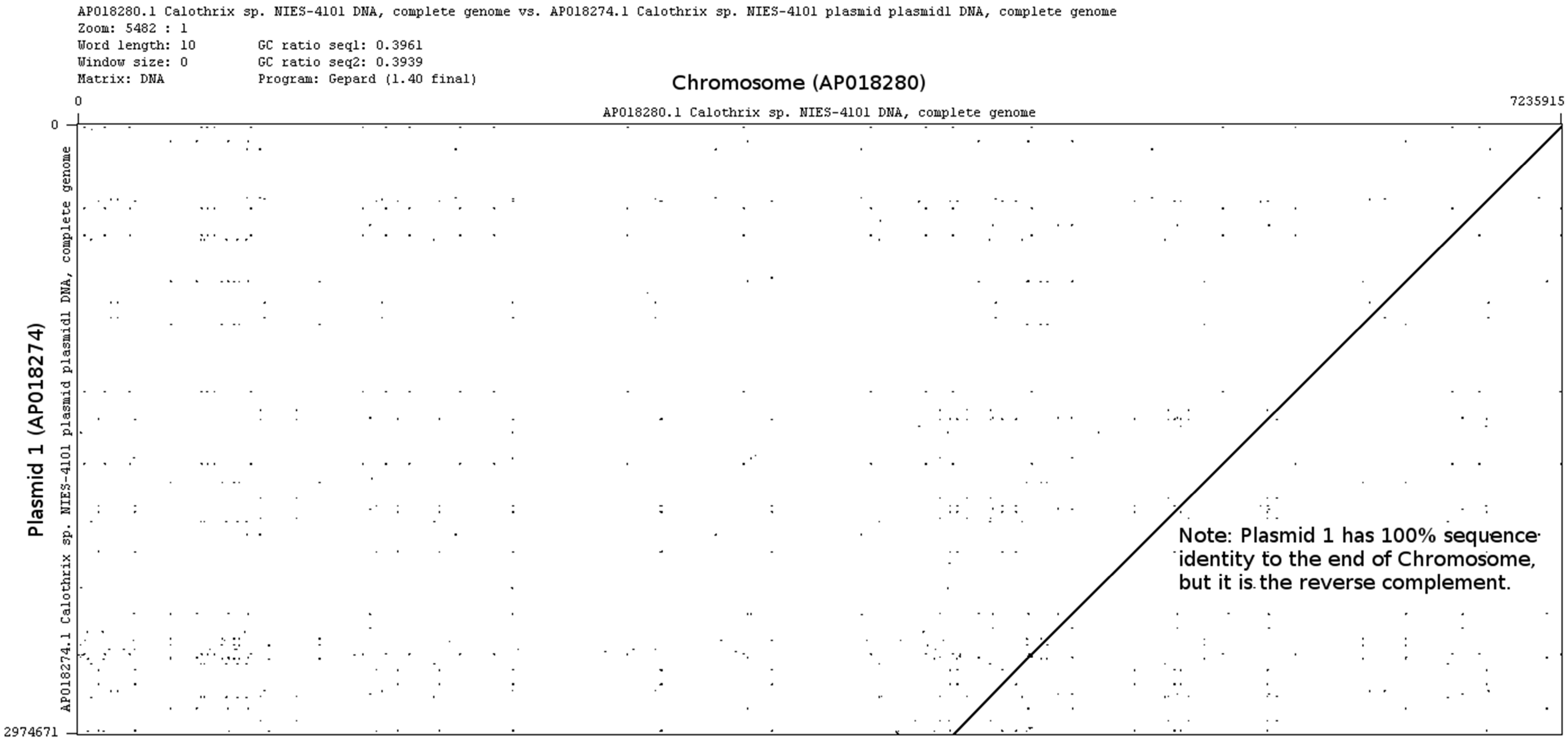

Supplementary Figure S4. A Gepard dotplot (Krumsiek,J., Arnold,R. and Rattei,T. (2007) Gepard: a rapid and sensitive tool for creating dotplots on genome scale. Bioinformatics, 23, 1026–1028.) showing the alignment of the chromosome (AP018280.1) and plasmid 1 (AP018274.1) of the genome assembly of Calothrix sp. NIES-4101. Of note, the sequence of plasmid 1 has 100.0% sequence identity to the end of the chromosome, but is the reverse complement.

5. Supplementary Table S1

Supplementary Table S1. Overview of the most repeat-rich *Pseudomonas* genomes. Twelve strains with the highest overall number of repeats are shown.

Organism	Strain	Accessions	Number of repeats	Longest repeat	Isolation source
Pseudomonas syringae pv. *actinidiae*	ICMP 9853	CP018202.1,CP018203.1,CP018204.1	587	28270	Kiwifruit (*Actinidia sp.*)
Pseudomonas syringae pv. *actinidiae*	NZ-45	CP017007.1,CP017008.1	489	13099	Kiwifruit (*Actinidia deliciosa*)
Pseudomonas syringae pv. *actinidiae*	NZ-47	CP017009.1,CP017010.1,CP017011.1	478	8961	Kiwifruit (*Actinidia chinensis*)
Pseudomonas syringae pv. *actinidiae*	CRAFRU 14.08	CP019732.1,CP019733.1	473	8960	Kiwifruit (*Actinidia deliciosa*)
Pseudomonas syringae pv. *actinidiae*	CRAFRU 12.29	CP019730.1,CP019731.1	471	8961	Kiwifruit (*Actinidia deliciosa*)
Pseudomonas syringae pv. *actinidiae*	ICMP 18708	CP012179.1,CP012180.1	469	8961	Kiwifruit (*Actinidia sp.*)
Pseudomonas cerasi	NA	LT222319.1,LT222313.1,LT222314.1,LT222315.1,LT222316.1,LT222317.1,LT222318.1	459	20227	Sour cherry (*Prunus cerasus*)
Pseudomonas savastanoi pv. *phaseolicola*	1448A; BAA-978	CP000058.1,CP000059.1,CP000060.1	443	5874	Beans (*Phaseolus vulgaris*)
Pseudomonas aeruginosa	VRFPA04	CP008739.2	411	22445	Human
Pseudomonas syringae pv. *actinidiae*	ICMP 18884	CP011972.2,CP011973.1	408	8961	Kiwifruit (*Actinidia deliciosa*)
Pseudomonas syringae pv. *tomato*	DC3000	AE016853.1,AE016855.1,AE016854.1	290	7544	Tomato
Pseudomonas syringae pv. *actinidiae*	MAFF212063	CP024712.1,CP024713.1,CP024714.1	277	8956	Kiwifruit (*Actinidia chinensis*)

Pseudomonas syringae pv. *actinidiae* cluster in Figure 1B

**Isolation source:**
Plant
Human

6. Supplementary Table S2

Supplementary Table S2. Different assembly stages compared to the final Illumina-polished assembly.

	Flye assembly	After 3 Racon runs	After Nanopolish	After Illumina polishing
**Indels (per 100 kb) ***	919.61	389.28	125.45	0
**Mismatches (per 100 kb) ***	33.81	82.93	12.38	0
**Length of individual contigs**				
Chromosome	6,504,089 bp	6,472,521 bp	6,449,720 bp	6,444,290 bp
Plasmid 1	472,591 bp	469,698 bp	467,909 bp	467,568 bp
Plasmid 2	303,668 bp	303,281 bp	301,482 bp	300,131 bp
Plasmid 3	287,148 bp	285,372 bp	283,637 bp	283,378 bp
Plasmid 4	2,827 bp	2,827 bp	2,827 bp	2,827 bp
**Total**	**7,570,323 bp**	**7,533,699 bp**	**7,505,575 bp**	**7,498,194 bp**

* In relation to the final assembly, polished with Illumina MiSeq

7. Supplementary Table S3

Supplementary Table S3. Mapping statistics of reads from all three sequencing technologies to the final Illumina-polished assembly. Detailed information about the methods to generate the mapping statistics can be found in the code listing (see separate text file in the Supplementary Material).

Sequencing technology	Mapping reads in % on final assembly [*1]	Coverage on final assembly [*2]
Illumina MiSeq 2 x 300 bp (quality filtered)	99.97%	97-fold (stddev: 39)
PacBio (quality filtered, reads above 500 bp)	99.60%	298-fold (stddev: 86)
Oxford Nanopore (quality filtered, reads above 30 kb)	98.53%	159-fold (stddev: 46)

[*1] Mapping ratio based on quality filtered data for all sequencing technologies. Applying quality filtering to the raw data of ONT, PacBio and Illumina results in a mapping ratio of nearly 100% of the reads from each technology. Since quality filtering is agnostic with regard to the source of the sequencing reads, this is a simple way to check if an assembly likely is complete.
[*2] Coverage information based on unfiltered data. This is roughly the coverage that was used by the assemblers.

8. Supplementary Code Listing

Supplementary Code Listing. Overview of the commands and the respective steps that were followed in order to arrive at the final *de novo* genome assembly of *P. koreensis* P19 E3. The version of the respective software tools used is provided as well, allowing to reproduce the genome assembly. In addition, the steps to generate the mapping information in Supplementary Table S3 are provided.

The detailed Supplementary Code Listing is provided as an additional text file (formatted as text file with Linux new lines).

9. Supplementary Table S4

Supplementary Table S4. Repeat analysis overview for 9331 bacterial and 293 archaeal complete genomes. The classification for genome assembly complexity is also given. Genomes without repeats longer than 500 bp and a similarity higher than 95% (original cut-offs used by Koren and colleagues (Koren S et al. (2013) Reducing assembly complexity of microbial genomes with single-molecule sequencing. Genome Biol., 14, R101) were binned and listed as having no repeats. Genomes are sorted in descending order based on their longest repeat. We kindly ask users to reference our paper.

Due to its large size, Supplementary Table S4 is provided as an additional Excel-file.

10. Supplementary Table S5

Supplementary Table S5. Overview of the mappability of long ONT reads in different size bins. Very long reads can represent erroneous signals. Some of those "reads" are actually not real DNA molecules passing the pore of the MinION device but mostly something which is stuck in the pore and gets the algorithm to recognize a false positive very long read is passing, since the pore is occupied all the time and no "free pore" signal occurs. These reads are characterized by a very repetitive pattern and mostly have a very low quality value. See e.g. a blogpost by Prof. Nick Loman explaining this observation (http://lab.loman.net/2017/03/09/ultrareads-for-nanopore/).

Filtered length	Total reads	Mapped reads	Percent mapped reads
> 30 kb	34,167	30,412	89%
> 100 kb	386	171	44.3%
> 300 kb	25	2	8%

Reads were mapped with minimap2 v.2.5, option "-x map-ont".
